# Validation of the Arabic Version of the Patient Activation Measure (PAM-13) for Application within the Primary Healthcare Context in Saudi Arabia

**DOI:** 10.3390/healthcare11233090

**Published:** 2023-12-04

**Authors:** Ali Kerari, Malak Almalki, Ghareeb Bahari, Manal F. Alharbi

**Affiliations:** 1 Nursing Administration and Education Department, College of Nursing, King Saud University, Riyadh 11421, Saudi Arabia; alikariri@ksu.edu.sa; 2 Medical Physics Department, King Saud University Medical City, Riyadh 11472, Saudi Arabia; malmalki@ksu.edu.sa; 3 Maternal & Child Health Nursing Department, College of Nursing, King Saud University, Riyadh 11421, Saudi Arabia; maalwahbi@ksu.edu.sa

**Keywords:** patient activation measure, activation, validation, primary care

## Abstract

Background: Empowering patients with behavioral issues or chronic conditions to actively participate in their healthcare can help improve health outcomes. However, in the Saudi Arabian context, evaluation tools for achieving this goal are lacking, considering cultural and healthcare system factors. Therefore, this study aimed to determine the psychometric properties of the Arabic version of the Patient Activation Measure. Methods: This cross-sectional validation study was conducted on a sample of 225 patients receiving treatment from primary healthcare centers located in Riyadh, Saudi Arabia. Item analyses and reliability and construct validity testing of the tool were conducted. Results: The item–total correlation coefficients ranged from 0.31 (item 2) to 0.57 (item 11). The item–total correlation coefficients for all 13 items were above 0.30. The reliability was 0.80. A two-factor model (“knowledge and beliefs” and “confidence and skills”) reflecting the instrument was constructed. The raw model did not sufficiently fit the data (χ^2^ = 170.98, degree of freedom (df) = 64, *p* < 0.001; Tucker–Lewis index (TLI) = 0.79; comparative fit index (CFI) = 0.83; root mean square error of approximation (RMSEA) = 0.86 [90% confidence interval {CI} = 0.07–0.10]). After all significant correlations between the items’ error terms were modeled, an adequate fit was achieved (χ^2^ = 76.76, df = 51, *p* < 0.01; TLI = 0.94; CFI = 0.96; RMSEA = 0.04 [90% CI = 0.02–0.07]). Conclusions: the Arabic version of the Patient Activation Measure can be utilized by healthcare providers to assess the activation levels and unique needs and preferences of Arabic-speaking individuals and tailor interventions accordingly to provide necessary support.

## 1. Introduction

As people’s knowledge and understanding expand, they become more actively engaged in their own treatment and strive to ensure that healthcare services align with their specific needs and preferences [1]. Involving people in healthcare plans can help promote better health outcomes, including quality of care and patient satisfaction. Individuals with pre-existing medical conditions typically expect that their healthcare providers will keep them informed about any new research findings or technological advancements relevant to their specific conditions [2]. Healthcare providers should also adopt a patient-centered approach to care, including actively engaging patients in their care planning [3]. Patient activation refers to people’s knowledge, skills, confidence, and motivation in managing their own health. It is the process by which patients become actively involved in their care, make effective decisions, and take actions to improve their health outcomes [4].

It is important to recognize that every patient is an individual who has their own set of needs. It is the responsibility of healthcare staff to fulfill the needs of patients as individuals. This goal can be achieved by listening to patients’ concerns, preferences, and goals and involving them in the decision-making process [5]. A large number of patients engaged in their healthcare report improved emotional, physical, and social health outcomes [6]. Active participation of patients in their own healthcare, including decision making, goal setting, and self-care, is essential to improve the quality of care. Activated patients are usually proactive in seeking health information, asking relevant questions, and collaborating with healthcare professionals to actively participate in their care plans and self-management of their health conditions [7].

In some countries, such as Saudi Arabia, chronic conditions including hypertension, diabetes, and asthma are prevalent [8]. Patient involvement in care plans has been gaining recognition as an important aspect of the healthcare for patients with chronic conditions [9]. Efforts are being made to empower patients to actively participate in their own care, make effective decisions, and take responsibility for their health outcomes [10]. Initiatives, such as educational programs, digital health tools, and patient engagement strategies, are being implemented to foster patient care and improve overall healthcare experiences in the country [11]. The promotion of patient activation is seen as a key component in achieving patient-centered care and enhancing the overall quality of healthcare services [12]. Further, empowering patients with behavioral issues to become actively engaged in their healthcare can help improve treatment outcomes [13]. Strategies such as personalized education, tailored communication approaches, and shared treatment decision making can enhance patient activation in such specific populations, leading to improved overall healthcare experiences and better health outcomes [14].

The Patient Activation Measure (PAM) is a tool used to evaluate patients’ level of knowledge, skills, confidence, and ability to effectively manage their chronic medical conditions independently [15]. The tool was developed according to the following steps: devising the framework, determining the measurement approach, developing the original 22 items, pilot testing its effectiveness on people with or without chronic conditions, and refining and finalizing the tool. Next, the items were reduced to 13 questions deemed to be contributing meaningfully to the measurement [16].

Previous studies have evaluated the performance of the PAM-13 among patients with multiple health conditions such as those requiring lumbar spine surgery [17], diabetes [18], and human immunodeficiency virus [19]. These health conditions have been selected for several reasons. First, they represent diverse chronic conditions that significantly impact individuals’ health and require ongoing management. Second, these conditions have been the main focus of previous research validating the PAM-13, making them relevant examples to show the tool’s reliability and validity. Further, the three medical conditions have varying degrees of complexity and self-management demands, demonstrating an effective utility of the PAM-13 in assessing patients’ preparedness and involvement in managing their health conditions. The prevalence of these conditions differs according to several factors, such as geographic location, lifestyle, access to healthcare, and other potential preventive measures. The utility of the PAM-13 in these patient populations has shown promising results in measuring their knowledge, skills, confidence, and overall engagement in managing their conditions.

Compared with other existing measurement tools for patient activation, the PAM-13 provides a more nuanced understanding of patients’ abilities, empowering healthcare providers to tailor interventions and support patients accordingly [20]. This tool also supports patients’ self-management behaviors and outcomes in the context of chronic conditions [21]. Further, its brevity and reliability make it a practical and easily self-administered instrument for the intended population. The PAM-13 has been translated into several languages, including Arabic [21]. Owing to the impact of the recent COVID-19 pandemic, Al-Juffali and colleagues were unable to assess certain crucial factors in their study [22]. Further, patients’ needs and circumstances are distinct and require specialized assessment; therefore, additional evaluations are required to obtain more comprehensive results that can cater to a wider range of patients’ needs.

It is essential to implement a reliable and valid evaluation methodology to assess patient activation and understand the level of knowledge, skills, confidence, and engagement in self-management among individuals. A comprehensive search for instruments to measure patient activation in the self-care of chronic conditions, specifically in Saudi Arabia, did not yield any relevant instruments. The absence of such tools highlights the need for the development or adaptation of a measurement scale tailored to the Saudi Arabian context, considering cultural and healthcare system factors. Therefore, this study aimed to determine the psychometric properties of the Arabic version of the PAM-13 (PAM-13-A) in Saudi Arabia. The results are expected to contribute to enhancing the quality of patient care and addressing any areas where improvements are needed.

## 2. Materials and Methods

### 2.1. Study Design and Setting

A cross-sectional validation design was adopted to evaluate the applicability of the PAM-13 within the primary care context in Saudi Arabia. The study was conducted at primary healthcare centers (PHCs) located in Riyadh, the capital city of Saudi Arabia and the largest and fastest developing city in the country, primarily owing to their feasibility and convenient accessibility. PHCs in Saudi Arabia are managed by the Ministry of Health. They provide a range of primary care services, including preventive care, diagnosis, treatment, and management of common health conditions. PHCs are the initial point of contact for Saudi people seeking healthcare, contributing to improvements in patient activation and health outcomes [23]. In addition to their primary role, PHCs offer various other services to meet the diverse needs of individuals and regions throughout the country. These services include managing common chronic conditions, addressing specific healthcare requirements of different regions, and catering to the unique needs of individuals. The centers also provide routine check-ups, administer vaccinations, and facilitate referrals to specialized care whenever necessary [24].

### 2.2. Ethical Considerations

The study was approved by the Institutional Review Board of King Saud University (reference number: KSU-HE-22-617). Informed consent was obtained from all participants. The participation was completely voluntary, and the participants had the right to withdraw from the study at any time without penalty.

### 2.3. Sampling

Convenience sampling was used to recruit people who were readily available and accessible and met the general inclusion criteria. Saudi adults visiting PHCs for treatment were eligible for participation. Patients seeking care at PHCs are often considered suitable for evaluation, as they provide insights into proactive involvement in maintaining their well-being and preventing and treating chronic conditions [25]. Those with a fluent understanding of the Arabic language were also included, as the Arabic translated version of the questionnaire was used. Those who had been recently diagnosed with a chronic disease were excluded, as limited experience in self-management of chronic conditions may significantly confound the research outcomes. Individuals with cognitive impairment were also excluded, as they are likely unable to provide accurate and reliable responses. The sample size was calculated using a suggested number of 5–10 participants per item when running a confirmatory factor analysis (CFA) [26,27]. Since the PAM-13 contains 13 items, this study required a minimum sample size of 130 respondents. Accordingly, a total sample size of 225 participants was included, which was appropriate to assess the instrument’s psychometric properties.

### 2.4. Instrumentation

The participants were invited to complete an online survey that assessed their general sociodemographic and clinical information, including age, sex, educational level, and presence of a chronic illness.

The PAM-13 was used to assess the following three main components: self-reported knowledge, motivation, and skills for self-management. The use of the PAM-13-A was authorized by Insignia Health, LLC. (Portland, OR, USA). The 13 items are rated on a 4-point Likert scale (1 = strongly disagree to 4 = strongly agree). Stage 1 (being disengaged and unprepared) is measured with items 1 and 2; stage 2 (being aware but struggling), items 3–8; stage 3 (taking necessary actions), items 9–11; and stage 4 (maintaining a behavior), items 12 and 13. Activation scores are calculated by summing the responses to the 13 items, with higher scores indicating increased patient engagement in healthcare services and self-management activities. Permission to use the PAM-13 was obtained by the second author.

### 2.5. Data Collection Procedures

Data were collected using the online questionnaire. Online questionnaires are typically administered using a web-based platform that allows participants to complete the form using a computer, tablet, or smartphone. Such method is also cost-effective and efficient, and researchers can reach a large number of participants rapidly and easily [28]. After all required approvals were obtained, a research team member approached the PHCs’ administration to explain the study and request the recruitment of participants who met the inclusion criteria. Word of mouth and personal references were also utilized as effective means to facilitate data collection.

In the created link address, the participants could access information about the study (i.e., aims, data collection procedures, informed consent, and contact information). The participants who wished to participate in the study provided their consent by clicking the “Agree” button, after which they started filling out the questionnaire. Upon completion of the questionnaire, the participants had the option to submit it automatically to the researchers.

Identifiable information was not collected to ensure that patient identities were protected throughout the research. No confidential coding was required, as the participants independently completed the questionnaire without any external influence. Additionally, secure data storage and access protocols were implemented, limiting access to the research team members only. These measures were applied to safeguard the confidentiality and anonymity of the data.

### 2.6. Data Analysis

The SPSS (V. 28, IBM Corp., Armonk, NY, USA) and Analysis of Moment Structures (V. 28) were used for the data analysis, considering a significance level of 0.05. Descriptive statistics included means, standard deviations (SDs), numbers, and percentages. The reliability and validity of the PAM-13-A were assessed. Item analyses were performed using item–total correlation coefficients. As the PAM-13 is a unidimensional Guttmann-like instrument, it was used to assess the three main components: self-reported knowledge, motivation, and skills for self-management [16,29,30]. Thereafter, Cronbach’s α, which is the most often used parameter to assess reliability in similar validation studies of the scale, was calculated [31,32] to evaluate the PAM-13-A’s internal consistency. Similar to Cronbach’s alpha, McDonald’s omega coefficient was also evaluated. In addition, McDonald’s omega (ω) is used to assess multidimensional scales’ reliability because this method is more accurate [33,34]. Values of 0.70 or above were considered acceptable. The construct validity of the scale was assessed using exploratory factor analysis (EFA) and CFA.

Important test values were highlighted during the EFA and factor extraction. First, the Kaiser–Meyer–Olkin (KMO) value was calculated to examine the adequacy of the sample size. A KMO value larger than 0.5 was considered appropriate to proceed with factor analysis. Bartlett’s test of sphericity was also conducted to evaluate the factor model’s appropriateness. A significant Bartlett’s test of sphericity value was considered necessary to proceed with EFA. These test values were assessed in the first step. Second, the PAM-13-A’s dimensionality and construct validity were evaluated via EFA using principal component analysis (PCA) with oblique rotation. Factors with an eigenvalue larger than 1 were extracted, and a factor loading larger than 0.30 was set as the lower cutoff point in the formation of the factor structure [35].

CFA was conducted to assess the construct validity of the PAM-13-A. Significant correlations between the items’ error terms were appropriately modeled after relevant modification indices. The goodness-of-fit indices were evaluated in accordance with the Hu and Bentler criteria: Tucker–Lewis index (TLI) of ≥0.95, comparative fit index (CFI) of ≥0.95, and root mean square error of approximation (RMSEA) of ≤0.06 (upper 90% confidence interval [CI] limit of ≤0.08). The χ^2^ test was also used [35,36].

## 3. Results

### 3.1. Sample Characteristics

Table 1 summarizes the demographic characteristics of the participants. The average age of the participants was 53 years (SD = 12.5). Approximately 68% of the sample (n = 152) identified as women. Nearly 40% of the participants reported living with a chronic condition. Further, the majority of the sample (71.6%) had completed education beyond high school. The mean PAM-13-A score was 64.11 (SD = 14.74).

### 3.2. Questionnaire’s Reliability

The total scale of the 13 items obtained a total ω coefficient = 0.80, which indicates good overall internal consistency. The item–total correlation coefficients ranged from 0.31 (item 2) to 0.57 (item 11). The item–total correlation coefficients for all 13 items of the PAM-13-A were larger than 0.30. The reliability of the PAM-13-A is further detailed in Table 2.

### 3.3. Questionnaire’s Construct Validity

The PCA of the PAM-13-A revealed adequate data for the analysis (KMO value = 0.82; χ^2^ = 674.23, degree of freedom (df) = 78, *p* < 0.001) (Table 3). Two components of the PAM-13-A with an eigenvalue larger than 1 were extracted from the EFA: Components 1 and 2 explained 30.89% and 20.81% of the variances, respectively (Table 4). Component 1 (items 1–4), with factor loadings larger than 0.53, was labeled “knowledge and beliefs”. Component 2 (items 5–13), with factor loadings larger than 0.49, was labeled “confidence and skills”. The estimated correlations among factors were reported at 0.45.

**Figure 1:**
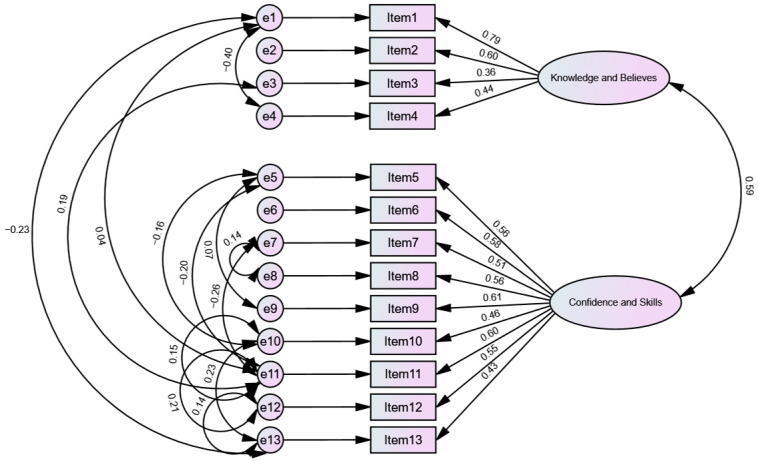
CFA of the two-factor model of the PAM-13.

Based on the EFA findings, CFA was run to determine the construct validity of the PAM-13-A using structural equation modeling. A two-factor model (“knowledge and beliefs” and “confidence and skills”) reflecting the PAM-13-A was constructed. The raw model did not adequately fit the data (χ^2^ = 170.98, df = 64, *p* < 0.001; TLI = 0.79; CFI = 0.83; RMSEA = 0.86 [90% CI = 0.07–0.10]). After all significant correlations between the items’ error terms were modeled, an adequate fit was achieved. The goodness-of-fit indices were satisfactory based on the Hu and Bentler criteria: (χ^2^ = 76.76, df = 51, *p* < 0.01; TLI = 0.94; CFI = 0.96; RMSEA = 0.04 [90% CI = 0.02–0.07]). Further results are shown in Figure 1.

## 4. Discussion

This study evaluated the use of the PAM-13-A among patients with chronic conditions in Riyadh, Saudi Arabia. The scale has consistently been reported as a valid and reliable instrument in various validation studies, encompassing heterogeneous groups of individuals with diverse chronic illnesses worldwide. It is an appropriate tool for measuring patients’ knowledge, skills, and confidence relative to the self-management of chronic conditions. To the authors’ knowledge, the present study is the first to test the validity of the PAM-13-A for use at PHCs in Saudi Arabia. The item analyses revealed a significant correlation between the PAM-13-A items and the overall scale. Generally, item–total correlation coefficients above 0.30 are considered acceptable, with coefficients exceeding 0.50 being recommended for instruments. The item–total correlation coefficients of the PAM-13-A fell within the recommended range, indicating satisfactory relationships between the individual items and the overall scale [35].

The mean PAM-13-A score among the patients in this study was 64.11 (SD = 14.74). This score is comparable to the mean activation score of 66.70 (SD = 12.3) observed among patients with metabolic syndrome visiting PHCs in Singapore. In contrast, lower mean activation scores (60.20 [SD = 12.7]) were observed among patients with chronic conditions in Brazil [37]. Further, the mean activation score in a sample of Germans under outpatient mentoring was 68.3 (SD = 14.8), higher than that reported in the present study. The variations in average activation scores observed between the mentioned studies and our study might be attributed to the inclusion of different age groups.

During the validation of an instrument, evaluating reliability is essential. In this study, the PAM-13-A demonstrated satisfactory internal consistency (reliability) with a McDonald’s omega value of 0.80. The Cronbach’s alpha indicated a similar pattern. The item–total correlation coefficients ranged from 0.31 to 0.57. This finding is consistent with those reported for translated versions of the PAM-13 into other languages: Italian (Cronbach’s α = 0.88) [38], Dutch (Cronbach’s α = 0.88) [20], Singaporean (Cronbach’s α = 0.86) [39], Brazilian (Cronbach’s α = 0.83) [40], American (Cronbach’s α = 0.81) [32], Turkish (Cronbach’s α = 0.81) [41], Malay (Cronbach’s α = 0.79) [31], German (Cronbach’s α = 0.79) [42], Spanish (Cronbach’s α = 0.93) [43], and Chinese (Cronbach’s α = 0.92) [44]. The existence of translated versions of the PAM-13 into other languages underscores its significance and relevance for patients with chronic conditions.

The concept of patient activation encompasses four domains: knowledge, beliefs, confidence, and skills required for maintaining appropriate disease self-management. These domains indicate four progressive levels of patient activation. The EFA and CFA in the current study revealed a two-factor solution for the PAM-13-A, explaining 51.7% of the variances. Conceptually, factor analyses support the original four-factor structure of activation proposed [45]. For example, the two-factor structure was thought to reflect a combination of two components from the original four-factor structure of activation. Component 1 was labeled as “knowledge and beliefs”, as it encompassed items 1–4, representing PAM-13 activation stages 1 and 2. Component 2 was labeled as “confidence and skills”, as it included items 5–13, reflecting PAM-13 activation stages 3 and 4. These components were identified, as they were conceptually congruent with the PAM model derived from the original USA validation data.

Consistent findings were also noted in a previous study on the factor validity of the Norway version of the PAM-13 [46]. The authors revealed two factors for the PAM-13, explaining 48.07% of the variances. Further, a quantitative study conducted in Iran reported a three-factor model, explaining 57.95% of the variances [47]. Another prior study also proposed a one-factor structure for the PAM-13, supporting the notion of a single underlying construct measured by the tool [31]. These differences across studies may be attributed to variations in sampling methods, participant responses, and cultural factors. Although there are minor differences between the previously mentioned versions of the PAM-13 and the PAM-13-A employed in the present study, the validated PAM-13-A demonstrated good applicability within the healthcare context in Saudi Arabia.

### 4.1. Limitations of the Study

This study provides evidence supporting the utility of the PAM-13-A as a valuable tool for measuring activation levels among patients in Saudi Arabia. However, certain limitations should be acknowledged. First, the study did not assess test–retest reliability owing to concerns about resource limitations and potential participant burden or withdrawal [38]. Only the internal consistency and construct validity were evaluated relative to the study purpose. Assessing test–retest reliability in future research would help obtain more reliable findings. Second, incorporating a broader range of PHCs and diverse geographical areas within Saudi Arabia might have enhanced the generalizability and robustness of the results. However, a Rasch model test to evaluate the instrument properties was not performed owing to the need for a larger sample size. Third, the representative sampling approach was limited, and response bias could exist, as the participants may not fully represent the target population or could provide inaccurate responses [48]. It is important to consider these limitations when interpreting and generalizing the findings to a larger population.

### 4.2. Study Implications

Patient activation plays an important role in facilitating effective self-management of chronic conditions. This study offers scientific evidence supporting the reliability and validity of the PAM-13-A in measuring activation levels relative to the self-management of chronic conditions among individuals visiting PHCs in Saudi Arabia. Healthcare professionals can utilize this tool to obtain valuable insights into patients’ knowledge, skills, and confidence in self-managing their conditions. This tool can also help healthcare providers in assessing individuals’ motivation and abilities necessary for effectively managing chronic conditions. Such an assessment has the potential to enhance clinical outcomes, decrease readmissions, and foster greater patient involvement in treatment plans.

Further, given the healthcare requirements in Saudi Arabia, healthcare professionals should consider the cultural, contextual, or health literacy factors that may influence patient activation levels. It is necessary to address such factors to help enhance patient engagement and activation in healthcare. To further support the utility of the PAM-13-A in the country, healthcare professionals can use the obtained insights into patients’ activation levels as well as unique needs and preferences to tailor interventions accordingly, including educational programs, counseling, and goal-setting strategies to improve patients’ self-management skills and confidence.

## 5. Conclusions

The PAM-13-A is a valid, reliable, and easily administered tool that can be utilized by nurses and other healthcare professionals for assessing activation levels among Arabic-speaking people relative to the self-management of chronic conditions. Its effectiveness and precision in assessment make it an important tool for enhancing patient engagement and empowerment in healthcare. Integrating the PAM-13-A into clinical practice may enhance patient-centered care and promote a proactive approach to better self-management among people with chronic conditions. We suggest that future research includes diverse age groups and collect data on patients’ health outcomes. This would effectively determine the relationship between PAM-13-A scores and health outcomes. Researchers are also encouraged to examine the PAM-13-A impact on other factors related to successful chronic disease self-management, such as medication adherence and decision-making skills, and to conduct further testing of the instrument in other Arabic-speaking countries.

## Figures and Tables

**Table 1:** Characteristics of the participants (N = 225).

Variable	Mean (SD) or *n* (%)
Age (years)	53 (12.5%)
Gender	
Male	73 (32.4%)
Female	152 (67.6%)
Education	
≤High school	67 (42.5%)
>High school	87 (57.5%)
Chronic Disease	
Living with a chronic disease	90 (40%)
Living without a chronic disease	135 (60%)
PAM-13 scores	64.11 (14.74%)

Note: SD, standard deviation; PAM-13, Patient Activation Measure-13.

**Table 2:** Item analyses and reliability of the Arabic version of the PAM-13.

Item	Mean (SD)	Corrected Item-Total Correlation	McDonald’s Ω If Item Deleted
1	3.15 (1.13)	0.35	0.80
2	3.22 (0.99)	0.31	0.80
3	3.04 (1.06)	0.36	0.80
4	2.77 (1.25)	0.36	0.79
5	3.11 (0.95)	0.47	0.78
6	3.08 (1.09)	0.53	0.77
7	3.17 (1.01)	0.44	0.78
8	3.05 (1.03)	0.51	0.77
9	2.88 (1.09)	0.53	0.77
10	2.69 (1.35)	0.42	0.78
11	2.89 (1.19)	0.57	0.77
12	2.62 (1.32)	0.51	0.77
13	2.46 (1.34)	0.39	0.79
Ω Total			0.80

Note: SD, standard deviation.

**Table 3:** Exploratory factor analysis of the Arabic version of the PAM-13.

Item	Factor 1 *	Factor 2 **
1	**0.79**	0.14
2	**0.73**	0.20
3	**0.55**	0.21
4	**0.53**	0.11
5	0.25	**0.58**
6	0.13	**0.64**
7	0.08	**0.54**
8	0.03	**0.68**
9	0.23	**0.51**
10	0.14	**0.74**
11	0.26	**0.71**
12	0.17	**0.63**
13	0.24	**0.49**

Note: extraction method—principal factor analysis. Rotation method—oblique with promax. All loadings greater than 0.40 are in bold. * knowledge and beliefs; ** confidence and skills.

**Table 4:** Total variance explained in a PCA for the Arabic version of PAM-13.

	Initial Eigenvalues		
Components	Total	% of Variance	Cumulative %
1	4.01	30.89	30.89
2	2.71	20.81	51.70
3	0.88	6.78	58.48
4	0.80	6.37	64.85
5	0.72	5.71	70.56
6	0.66	4.85	75.41
7	0.60	4.43	79.84
8	0.56	4.21	84.05
9	0.52	3.97	88.02
10	0.45	3.51	91.53
11	0.40	3.20	94.73
12	0.38	2.84	97.57
13	0.33	2.43	100

Note: PCA, principal component analysis.

## Data Availability

Data are not shared due to privacy and ethical restrictions.
